# Classifying and Monitoring Primary Progressive Aphasia in the Greek Population: A “Mini Linguistic State Examination (MLSE)” Tool

**DOI:** 10.3390/medicina61111998

**Published:** 2025-11-07

**Authors:** Valentina Papadopoulou, Eleni Konstantinopoulou, Aikaterini Liapi, Chrissa Sioka, Ioannis Iakovou, Eleni Aretouli, Panagiotis Ioannidis

**Affiliations:** 1 Lab of Cognitive Neuroscience, School of Psychology, Aristotle University of Thessaloniki, 541 24 Thessaloniki, Greece; 2 2nd Department of Neurology, AHEPA University Hospital, Aristotle University of Thessaloniki, 541 24 Thessaloniki, Greece; 3 Department of Nuclear Medicine, University Hospital of Ioannina, 455 00 Ioannina, Greece; 4 2nd Department of Nuclear Medicine, AHEPA University Hospital, Aristotle University of Thessaloniki, 541 24 Thessaloniki, Greece; 5 Department of Psychology, School of Social Sciences, University of Ioannina, 451 10 Ioannina, Greece

**Keywords:** PPA, language impairment, MLSE, brain perfusion

## Abstract

*Background and Objectives*: Difficulties in language production and comprehension constitute clinical symptoms characterizing patients diagnosed with Primary Progressive Aphasia (PPA). Thorough assessment of language domains can detect specific deficits commonly observed in different PPA variants, but brief and practical instruments capable of screening for language impairment are lacking. The present study aimed to examine the ability of the Mini Linguistic State Examination (MLSE) to distinguish between healthy individuals and PPA patients, as well as to differentiate among PPA subtypes, within Greek clinical practice. *Materials and Methods*: Τhe Mini Linguistic State Examination (MLSE), a 15-min detailed examination of different language domains, was administered to a group of clinically diagnosed PPA patients and a group of healthy participants. In addition, PPA patients completed a neuropsychological test battery assessing memory, language, executive, and visuospatial functions. Patterns of patients’ brain perfusion were also explored with single-photon emission computed tomography. *Results*: Comparisons between PPA patients and healthy controls revealed significant differences across all MLSE domains (all *p* < 0.001), and receiver operating characteristic analyses demonstrated excellent diagnostic accuracy, with AUC values exceeding 0.90 across language domains and perfect classification for the total MLSE score (AUC = 1.00, *p* < 0.001). *Conclusions*: These findings indicate that the MLSE is able to detect distinct patterns of deficits and to provide a comprehensive overview of patients’ linguistic profiles, supporting its clinical utility and diagnostic potential for differentiating PPA variants.

## 1. Introduction

Primary Progressive Aphasia (PPA) is a clinical syndrome characterized by selective neurodegeneration of language networks and an evident progressive deterioration of linguistic ability, while other cognitive functions remain relatively preserved, at least at the initial stages of the disease [1,2,3]. Currently, the PPA syndrome is divided into three distinct clinical phenotypes: the semantic (svPPA), the non-fluent/agrammatic (nfvPPA) and the logopenic variant PPA (lvPPA) [2]. svPAA patients present with predominantly left anterior temporal lobe atrophy [4,5] and are primarily affected by severe impairment in semantic memory, exhibiting a limited vocabulary range, inability to categorize objects, and face and/or concepts recognition difficulty. Verbal communication is fluent but lacks content [6]. In nfvPPA variant, patients present with slow, hesitant verbal expression, marked by grammatical errors and simplification of speech, difficulty in writing sentences and, in a plethora of cases, apraxia of speech. Language comprehension remains relatively intact for simple grammatical structures, but it is impaired for grammatically complex sentences [7]. The aforementioned deficits are associated with structural and functional impairments of the left inferior frontal gyrus, insula and premotor/supplementary motor areas [5,8]. The lvPPA is characterized by frequent pauses in speech, due to difficulty in word retrieval and impaired phonological short-term memory. Prominent temporo-parietal and posterior perisylvian junction atrophy constitute its neuroimaging hallmark [9,10]. Patients retain grammatical structure and articulation but demonstrate deficits in repetition and processing of auditory information [2].

The key characteristics for detecting PPA and differentiating among its subtypes can be identified through neurological examination and detailed neuropsychological evaluation, using standardized measures for the assessment of the core language domains affected. In routine clinical practice in Greece, comprehensive tools are available for the diagnosis of aphasia, naming the Boston Diagnostic Aphasia Examination and the Minnesota Test for Differential Diagnosis of Aphasia [11], which, however, are not optimized for the differential diagnosis of PPA. They primarily assess a wide range of suspected language deficits, thus the evaluation of PPA patients relies on a time-consuming, informal, and qualitative assessment of the already identified and isolated difficulties. Patel et al. [12] recently introduced the Mini Linguistic State Examination (MLSE), a short, comprehensive bedside tool suitable for the direct detection of patterns of language impairments corresponding to the three PPA variants. In approximately 20 min, the MLSE, including 11 subtests, can provide the clinician with information for patient’s performance in five key language domains: (1) motor speech, (2) semantic knowledge, (3) phonology, (4) syntax, and (5) verbal working memory. The comprehensive assessment of these domains offers a distinct language profile for each of the three PPA variants, as well as an overall score that indicates the severity of the disease [12,13].

Recent studies have expanded the MLSE beyond its original English version, providing evidence for its cross-linguistic validity and clinical applicability. The Spanish adaptation of the MLSE demonstrated satisfactory internal consistency (Cronbach’s α = 0.758) and excellent discriminant ability (AUC ≈ 0.99) between PPA patients and healthy controls, with minimal influence of demographic variables [14]. In addition, a recent comparative investigation of MLSE, Addenbrooke’s Cognitive Examination (ACE-III) and Dépistage Cognitif de Québec (DCQ) reported strong diagnostic capacity for the MLSE (AUC ≈ 0.95) and reasonable accuracy for PPA variant classification, supporting the instrument’s utility across clinical samples [15]. Preliminary reports and pilot data have also described the intelligibility and feasibility of Portuguese-language implementations [16].

These cross-linguistic initiatives indicate that the MLSE’s structured, domain-based approach can be applied in diverse linguistic contexts, yet they also highlight well-documented challenges in adapting language-based neuropsychological tools across different populations [17,18,19]. Semantic and psycholinguistic equivalence should be sought carefully because lexical frequency, imageability and concreteness differ between languages and can influence construct validity. Morphosyntactic variability may alter the cognitive demands of syntax tasks, and phonological/articulatory differences may bias repetition or non-word tasks. Moreover, cultural familiarity with pictorial or verbal stimuli, along with demographic factors such as education and dialectal variations, necessitate systematic pilot testing and development of local normative data to ensure fair and accurate clinical interpretation.

In the Greek linguistic context, these aspects are particularly pertinent given the language’s morpho-syntactic complexity and regional variation. Rather than a formal adaptation, the present study therefore represents an initial implementation of the MLSE in Greek-speaking individuals. Beyond assessing its feasibility and clinical utility in distinguishing PPA patients from healthy controls, our main objective was to examine its capacity to differentiate between PPA subtypes. We further compared MLSE performance with standardized neuropsychological measures to evaluate its usefulness for clinical practice, while SPECT imaging was investigated to contextualize the behavioral findings within a neurobiological framework.

## 2. Materials and Methods

### 2.1. Participants

A total of twenty-eight patients (14 females) who satisfied the International Classification Criteria for PPA were recruited from a university-affiliated neurology clinic in Northern Greece. Diagnosis was based on a comprehensive neurological evaluation, including a structured medical interview, neurological examination, and neuropsychological assessment. The patients were further classified into the three main PPA variants according to established clinical criteria [9], yielding 11 lvPPA, 7 nfvPPA and 10 svPPA patients. Participants who did not meet the consensus criteria for PPA or presented with previous neurological and/or psychiatric history were excluded.

The control group included thirty-two healthy adults (19 females), with no past or present neurological and/or psychiatric diagnoses, and a Mini Mental State Examination (MMSE) total score greater than 26. Demographic and clinical characteristics of participants are presented in Table 1 and Table 2. For consistency with the existing MLSE literature, descriptive results are expressed as means and standard deviations, even though the data were non-normally distributed.

All participants were native Greek speakers and provided informed consent in accordance with the Declaration of Helsinki ethical guidelines. The study protocol was approved by the Ethics Committee of the Medical Department of Aristotle University of Thessaloniki.

**Figure 1:**
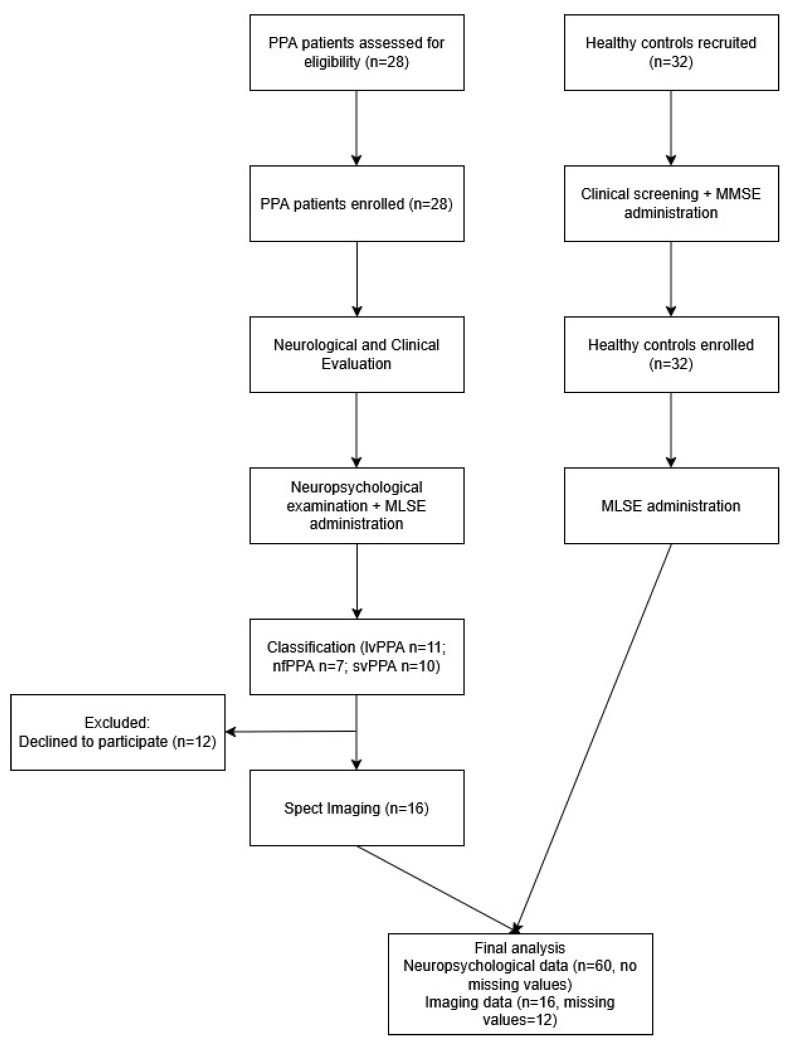
Participant flow diagram displaying the progression of participants through screening, enrollment, examination and analysis.

*Participant flow*: Initially, 28 suspected PPA patients and 32 healthy controls were screened for eligibility. Of the patients, 12 declined to undergo neuroimaging due to personal reasons. Therefore, 28 patients and 32 controls meeting the inclusion criteria were enrolled and completed the neuropsychological assessment. All participants were included in the final statistical analyses of clinical and cognitive data, with no missing values. Neuroimaging analyses were conducted on the subset of patients who completed SPECT scanning (n = 16). A corresponding participant flow diagram is presented in Figure 1.

### 2.2. Measures

The MLSE was administered to all participants to assess performance in five language domains: (1) motor speech, (2) semantic knowledge, (3) phonology, (4) syntactic knowledge, and (5) auditory–verbal working memory. The test includes 11 subtests that evaluate performance in language components commonly affected in PPA: (1) picture naming, (2) syllable and multisyllable repetition, (3) word repetition and single-word comprehension, (4) non-word repetition, (5) semantic association, (6) sentence comprehension (verbal), (7) sentence comprehension (pictorial), (8) word and non-word reading, (9) sentence repetition, (10) writing, and (11) picture description. The subtests provide a distinct score per language domain, forming linguistic profiles that correspond to the PPA variants, as well as a total score offering information for disease severity.

In addition, all patients underwent a standardized neuropsychological assessment assessing major cognitive domains in accordance with the neurology clinic’s protocol for the evaluations of patients with neurodegenerative pathology: verbal learning and memory (Greek version of word learning test [20]), language and semantic memory (Object naming test), visuoperceptual ability (Judgment of Line Orientation [21,22] abbreviated form), attention and information processing speed (Trail Making Test (TMT) [23], working memory (Digit span Forward) and executive functioning (TMT-Part B; Digit span Backward). The MMSE was administered to ensure the inclusion of cognitively intact participants in the control group.

Finally, Single Photon Emission Computed Tomography (SPECT) scans were available for a subgroup of 16 patients. Imaging data acquisition, along with patient preparation and assessment, were performed in accordance with the EANM guidelines for brain perfusion SPECT studies [24]. Prior to scanning, participants were instructed to abstain from alcohol, caffeine, or other stimulants and substances that might alter cerebral blood flow (rCBF). SPECT scans were performed 30 min after intravenous injection of 740 MBq Hexamethyl Propylene Amine Oxime labeled with Technique-99 m, (^99m^Tc-HMPAO SPECT), while patients were at rest, with their eyes open and ears unplugged. The procedure was performed in a silent environment, minimizing any interaction with the participant. A single-headed gamma camera (Philips with Pegasys processor) was used, acquiring 120 projections per participant, each lasting 20 s (total duration: 40 min). Image reconstruction was performed in an XELERIS Workstation (Version 3.1) using filtered back projection (FBP) with a Butterworth filter (order 10, cutoff 0.5), realigned in coronal, sagittal, and transverse planes. The NeuroGam™ software was applied to the reconstructed dataset, enabling automated analysis of rCBF in both, cortical lobes and Brodmann areas, for the left and right hemispheres.

### 2.3. Data Analysis

Data distributions were assessed for normality using the Shapiro–Wilk test. Since most variables deviated from normality, non-parametric tests were applied. With regard to the MLSE performance, group comparisons among the three PPA groups (i.e., lvPPA, nfvPPA and svPPA) were performed using non-parametric statistics (Kruskal–Wallis test), followed by post hoc pairwise comparisons using Dunn’s test with Bonferroni adjustment for multiple comparisons where applicable. Receiver Operating Characteristic (ROC) curve analyses were conducted to evaluate the discriminative ability of the MLSE in distinguishing PPA patients from healthy controls and differentiating among PPA subtypes. Area Under the Curve (AUC) values were calculated as indicators of classification performance. The area under the curve (AUC) and corresponding 95% confidence intervals (CIs) were computed using the nonparametric asymptotic method (DeLong). Given the relatively small sample size, bootstrapping for CI estimation was not applied, as resampling from a limited dataset could yield unstable estimates.

The associations between MLSE domain and total scores and the standardized neuropsychological measures, were examined via non-parametric Spearman’s rho correlation. Given the limited sample size and the non-parametric nature of the data, confidence intervals for correlation coefficients were not computed, as they would provide imprecise estimates.

All statistical tests were two-tailed, with the significance threshold set at *p* < 0.05. To account for multiple comparisons, the False Discovery Rate (FDR) correction was applied using the Benjamini–Hochberg procedure.

To further explore regional perfusion patterns in patients diagnosed with PPA variants, standardized rCBF scores, extracted from NeuroGam^TM^ software (Segami-Corporation, Columbia, SC, USA, www.segamicorp.com, accessed on September 2025), were analyzed for a set of a priori defined regions of interest (ROIs) based on their established association with each PPA variant. For each ROI, a rCBF value of two standard deviations below mean expected perfusion (in accordance with the NeuroGam^TM^ normative values) was used as a threshold to indicate significant hypoperfusion. Given the small and unequally distributed group sizes (lvPPA = 7, nfvPPA = 3, svPPA = 6), formal statistical comparisons were deemed underpowered. Therefore, a descriptive approach was adopted, reporting the proportion of patients within each variant group who exhibited hypoperfusion in the target ROIs.

All analyses were performed using SPSS Statistics software (version 29.01).

## 3. Results

### 3.1. MLSE Diagnostic Accuracy

Analyses among PPA variants yielded significant performance differences across specific MLSE domains. Kruskal–Wallis tests showed significant effects for the Motor Speech (x^2^ = 9.64, *p* = 0.008, η^2^ = 0.36) and Phonological domains (x^2^ = 8.71, *p* = 0.013, η^2^ = 0.32). Bonferroni-adjusted post hoc analyses indicated that nfvPPA patients scored significantly lower than svPPA patients on both subscales, whereas no significant differences were identified between groups for the Semantic, Syntactic and Working memory domains (all *p* > 0.05), as presented in Table 3.

Group comparisons between PPA patients (n = 28) and healthy controls (n = 32) revealed significant differences across all MLSE domains. Controls performed at or near ceiling in phonology, syntax, and working memory, whereas patients exhibited pronounced impairments. All between-group comparisons reached statistical significance (all *p* < 0.001).

**Figure 2:**
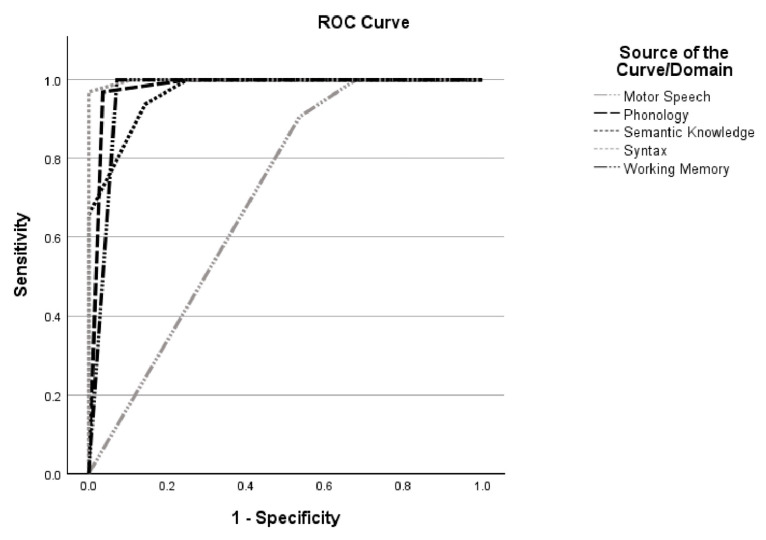
Receiver operating characteristics of the MLSE domains.

**Figure 3:**
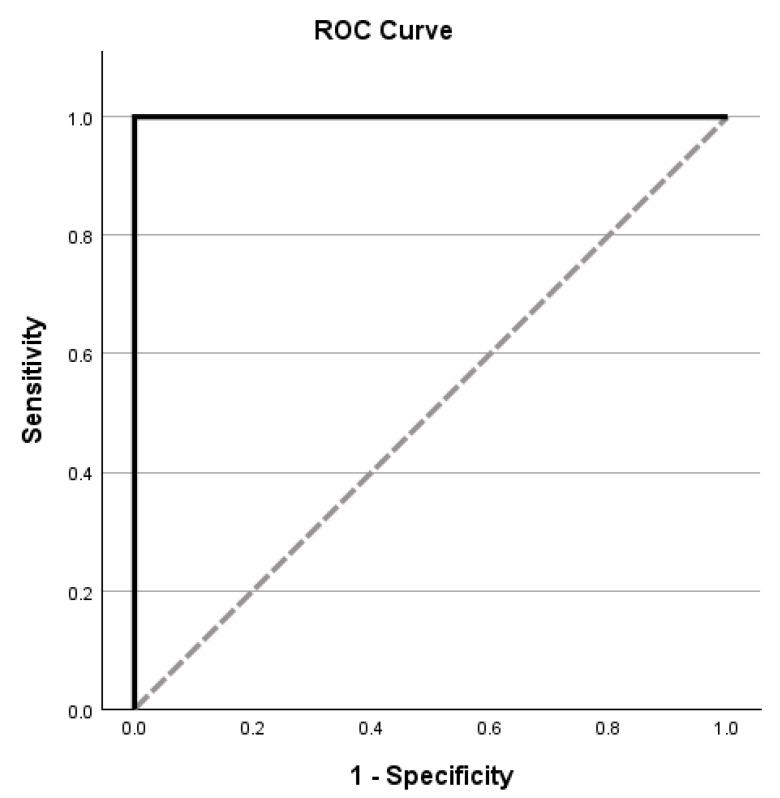
Receiver operating characteristics of the MLSE total score.

Receiver operating characteristic (ROC) analyses further demonstrated excellent diagnostic accuracy of the MLSE in distinguishing patients from controls. Area under the curve (AUC) values exceeded 0.90 for phonology, semantics, syntax, and working memory, with the syntactic domain showing near-perfect discrimination (AUC = 0.998, *p* < 0.001) [Figure 2]. Additionally, the total MLSE score achieved perfect classification between patients and controls (AUC = 1.00, *p* < 0.001) [Table 4; Figure 3]. In contrast, ROC analyses comparing PPA variants yielded unstable AUC values across domains, indicating limited, non-significant discriminative capacity for successful subtype classification in this sample.

### 3.2. Associations Between MLSE and Other Neuropsychological Measures

Spearman correlations indicated robust associations between MLSE performance and independent neuropsychological measures. Motor Speech domain correlated with working memory measures (r = 0.513, *p* = 0.006), while Phonological Processing domain was negatively related to processing speed (r = −0.540, *p* = 0.006) and positively to verbal working memory tasks (r = 0.470, *p* = 0.013). The Semantic domain was associated with verbal learning (r = 0.41, *p* = 0.034), visuospatial abilities (r = 0.420, *p* = 0.033), and showed a particularly strong link with the object naming task (r = 0.715, *p* < 0.001). Syntax was moderately associated with working memory (r = 0.405, *p* = 0.036). Moreover, the Working Memory domain and the MLSE Total Score demonstrated the strongest associations, being positively related to processing speed (r = 0.720, *p* < 0.001) and verbal working memory measures (r = 0.700, *p* < 0.001). Overall, higher MLSE performance was consistently linked to better memory, language, and executive functioning.

### 3.3. MLSE and Neuroimaging Findings

**Figure 4:**
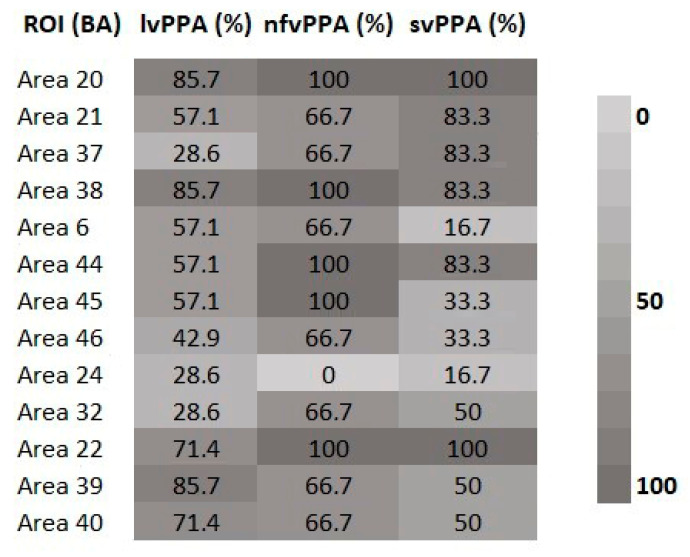
Proportion of patients with significant hypoperfusion (z ≤–2) in variant-specific ROIs across PPA subtypes. lvPPA = Logopenic variant of Primary Progressive Aphasia; nfvPPA = Non-fluent variant of Primary Progressive Aphasia; svPPA = Semantic variant of Primary Progressive Aphasia; BA = Broadmann Area; ROI = Region of Interest.

Descriptive analysis of SPECT data highlighted variant-specific patterns of hypoperfusion within the a priori selected ROIs. In the semantic variant, the most consistent findings involved the anterior temporal cortices with the majority of patients showing significant hypoperfusion in BAs 20 and 38 (85.7–100%), and frequent involvement of BAs 21 and 37. In contrast, nfvPPA patients demonstrated reduced perfusion in the inferior frontal regions, particularly BAs 44 and 45 (57.1–100%), while abnormal blood-flow changes in premotor and medial frontal areas (BAs 6, 24, 32, 46) were less consistent. In the logopenic variant, posterior temporal and inferior parietal cortices were most prominently affected, with high rates of hypoperfusion in BA 22 (71.4–100%), BA 39 (66.7–85.7%), and BA 40 (66.7–71.4%), alongside with more variable alterations in BAs 21 and 37. Although the small sample precluded formal statistical testing, these descriptive observations closely align with the expected neuroanatomical profiles of each PPA subtype. Detailed descriptive patterns for each ROI are summarized in Figure 4.

## 4. Discussion

The present study provides novel evidence for the applicability of the Mini Linguistic State Examination (MLSE) in the assessment and monitoring of Primary Progressive Aphasia (PPA) in a Greek-speaking cohort. Our results extend previous work [12,13] by demonstrating that the MLSE is highly sensitive in differentiating PPA patients from healthy controls, while also capturing meaningful variability across linguistic domains that correspond to the distinct clinical phenotypes of PPA. Consistent with earlier studies, patients with PPA showed pronounced impairments in motor speech, semantic knowledge, working memory and phonological and syntactic processing [7,9]. The MLSE detected these difficulties with high accuracy, supporting its clinical value as a brief yet comprehensive assessment tool. Importantly, the total MLSE score achieved perfect classification between patients and controls, mirroring prior findings in English-speaking populations [13] and indicating the robustness of the instrument across languages. However, its ability to discriminate among PPA variants was limited, with unstable AUC values across domains, possibly reflecting the modest sample size, overlapping linguistic deficits across variants, and the dynamic evolution of symptoms over disease progression. This limitation has been previously reported by Fernández-Romero and colleagues [15], where subtype classification accuracy ranged from 76% to 79%. Clinical heterogeneity and symptom evolution often blur subtype boundaries, rendering purely linguistic discrimination less reliable, particularly at early disease stages [25,26].

The correlations between MLSE domains and independent neuropsychological measures provide further construct validity. Motor Speech performance was linked to working memory capacity, while phonological processing was associated with processing speed and verbal working memory. Semantic and syntactic domains showed modest associations, aligning with evidence that such impairments may emerge later at the disease course or vary across individuals [27,28]. Notably, the strongest effects were found for the Working Memory domain and Total MLSE scores, which were robustly related to executive functioning performance. These findings highlight the interdependence between language, executive and memory processes in PPA [29,30] and support the MLSE’s ability to capture broader cognitive–linguistic interactions associated with daily communication.

With regards to neuroimaging data, the descriptive analysis of the rCBF findings revealed variant-specific hypoperfusion patterns consistent with previous literature. Participants diagnosed with svPPA showed greater hypoperfusion in anterior temporal cortices (particularly BAs 20 and 38), consistent with the documented anterior temporal lobe degeneration underlying semantic memory loss [6,31]. In addition, nfvPPA patients exhibited reduced perfusion in inferior frontal regions, including BAs 44 and 45, in line with prior structural and functional imaging studies implicating the left inferior frontal gyrus and premotor regions in speech apraxia and syntactic deficits [8,32]. Finally, lvPPA patients demonstrated prominent abnormalities in posterior temporal and inferior parietal areas (BAs 22, 39, 40). Previous studies have associated dysfunction in these regions with impaired phonological short-term memory and word retrieval [9,10], providing evidence that reduced integrity of posterior temporo-parietal cortices constitutes the functional substrate of lvPPA. At the same time, a subset of the predefined ROIs, showed less consistent and more variable patterns across patients (e.g., premotor and medial frontal regions in nfvPPA, or BA 37 in lvPPA). This variability is not unexpected. Similar findings have been reported in multimodal imaging studies [5,33], suggesting that regional involvement may depend on disease stage, methodological factors, or individual clinical trajectories. Τhe observed patterns closely match established neuroanatomical profiles, reinforcing their interpretive value despite the descriptive nature of the analysis.

Taken together, these results support the clinical utility of the MLSE as a brief, reliable instrument for assessing language impairment in PPA in Greek-speaking populations. The instrument is sensitive to both overall disease severity and domain-specific deficits, while the correlations with neuropsychological measures and alignment with neuroimaging findings reinforce its validity. However, difficulty in subtype classification highlights the need for a multimodal diagnostic approach, integrating language assessment with neuroimaging and biomarkers. Future research with larger samples and longitudinal designs should further investigate the MLSE’s association with disease progression and functional decline.

The present study has certain limitations that should be addressed. At first, the relatively small sample constrained statistical analyses and reduced power in detecting variant-specific differences. Secondly, the patient group included participants who voluntarily presented to the neurology clinic for scheduled neurological examination. As such, the cohort may not fully reflect characteristics of the broader PPA population. In addition, the cross-sectional design cannot provide evidence of how MLSE performance evolves through disease progression. Furthermore, only quantitative data from SPECT analyses processed via NeuroGam™ software were available, preventing the inclusion of representative SPECT images. This constitutes an additional limitation, as visual inspection of perfusion maps could further support the reported findings. Finally, the MLSE has not yet been standardized in Greek, therefore cultural and linguistic factors may influence performance in subtle ways, suggesting that cross-linguistic validation remains crucial. Accordingly, our study should be considered as a preliminary exploration of its clinical applicability rather than a normative standardization study.

## 5. Conclusions

In summary, this study offers the first exploratory application of the MLSE in a Greek-speaking PPA cohort. The instrument demonstrated excellent sensitivity in distinguishing patients from controls, meaningful associations with independent cognitive measures, and convergence with established neuroanatomical patterns of hypoperfusion in PPA. While its discriminative ability among PPA subtypes was limited, this finding is consistent with broader literature and underscores the inherent clinical complexity of PPA. Overall, the MLSE emerges as a valuable addition to a diagnostic toolkit, bridging the gap between comprehensive neuropsychological batteries and the need for rapid, reliable clinical assessment.

## Figures and Tables

**Table 1:** Demographic and clinical characteristics of PPA patients.

	Mean	Std. Deviation	Minimum	Maximum
Age				
Total (n = 28)	69.61	7.87	56.00	81.00
lvPPA (n = 11)	68.27	7.79	59.00	78.00
nfPPA (n = 7)	70.29	10.77	56.00	81.00
svPPA (n = 10)	70.60	6.09	57.00	76.00
Education				
Total (n = 28)	12.46	5.05	6.00	22.00
lvPPA (n = 11)	12.55	5.52	6.00	22.00
nfPPA (n = 7)	11.29	5.53	6.00	21.00
svPPA (n = 10)	13.20	4.54	6.00	22.00
Disease Duration				
Total (n = 28)	2.39	1.81	1.00	7.00
lvPPA (n = 11)	2.82	2.36	1.00	7.00
nfPPA (n = 7)	2.43	1.79	1.00	5.00
svPPA (n = 10)	1.90	0.99	1.00	4.00

lvPPA = Logopenic variant of Primary Progressive Aphasia; nfvPPA = Non-fluent variant of Primary Progressive Aphasia; svPPA = Semantic variant of Primary Progressive Aphasia.

**Table 2:** Demographic characteristics of healthy participants.

	Mean	Std. Deviation	Minimum	Maximum
Age	57.84	6.96	50.00	78.00
Education	15.03	2.66	12.00	21.00
MMSE Score	29.13	0.75	27.00	30.00

MMSE = Mini Mental State Examination.

**Table 3:** Results of Kruskal–Wallis analyses examining differences among PPA subtypes on the five distinct MLSE language domains and MLSE total score.

Domain	lvPPA	nfvPPA	svPPA	H(df)	*p*	η^2^	Post Hoc
	Median (IQR)	Median (IQR)	Median (IQR)				
Mot. Sp.	30.00 (4.00)	23.00 (13.00)	30.00 (0.25)	9.64(3)	0.008	0.36	svPPA > nfvPPA
Phon.	26.00 (8.00)	25.00 (3.00)	28.00 (2.00)	8.71(2)	0.013	0.32	svPPA > nfvPPA
Sem. Kn.	15.00 (6.00)	16.00 (6.00)	14.50 (6.00)	1.15(2)	0.562		
Synt.	5.00 (4.00)	5.00 (3.00)	6.00 (4.75)	2.66(2)	0.265		
Work. Me.	4.00 (7.00)	7.00 (3.00)	7.00 (2.50)	2.00(2)	0.368		
Total	82.00 (24.00)	72.00 (26.00)	83.50 (9.00)	3.64 (2)	0.162		

Mot. Sp. = Motor Speech; Phon. = Phonology; Sem. Kn. = Semantic Knowledge; Synt. = Syntax; Work. Me. = Working Memory; lvPPA = Logopenic variant of Primary Progressive Aphasia; nfvPPA = Non-fluent variant of Primary Progressive Aphasia; svPPA = Semantic variant of Primary Progressive Aphasia; IQR = Interquartile Range; df = Degrees of Freedom.

**Table 4:** Mini Linguistic State Examination (MLSE) Sensitivity and Specificity, and Area Under the Curve (AUC) scores with optimal cut-off scores for the differentiation of PPA patients and healthy controls.

MLSE Domains	AUC (95% CI)	Optimal Cut-Off	Sensitivity (%)	Specificity (%)
Motor Speech	0.700 (0.56–0.84)	≤29	90.6	47.1
Phonology	0.978 (0.94–1.00)	≤29	96.9	96.4
Semantic Knowledge	0.968 (0.93–1.00)	≤17	100.0	75.0
Syntax	0.998 (0.99–1.00)	≤9	96.9	100.0
Working Memory	0.964 (0.91–1.00)	≤9	100.0	92.9
Total Score	1.000 (1.00–1.00)	≤96	100.0	100.0

CI = Confidence Interval.

## Data Availability

The data that support the findings of this study are available from the corresponding author, upon reasonable request.

## Abbreviations

The following abbreviations are used in this manuscript:

PPA	Primary Progressive Aphasia
MLSE	Mini Linguistic State Examination
svPPA	Semantic variant of Primary Progressive Aphasia
lvPPA	Logopenic variant of Primary Progressive Aphasia
nfPPA	Non-fluent/agrammatic variant of Primary Progressive Aphasia
MMSE	Mini Mental State Examination
SPECT	Single Photon Emission Computed Tomography
rCBF	Cerebral blood flow
ROI	Region of Interest
BA	Brodmann Area
ROC	Receiver Operating Characteristic
AUC	Area Under the Curve
FDR	False Discovery Rate
